# Large Bowel Gallstone Ileus: Rare Spontaneous Passage Through a Cholecystocolonic Fistula

**DOI:** 10.7759/cureus.79754

**Published:** 2025-02-27

**Authors:** Daniel Hahn, Jonathan Kestenbaum, Julia Speiser, Katherine J Hahn

**Affiliations:** 1 Internal Medicine, Touro College of Osteopathic Medicine, New York, USA; 2 Gastroenterology, Cedars-Sinai Medical Center, Los Angeles, USA

**Keywords:** biliary disease, cholecystocolonic fistula, gallstone ileus, gallstones, large bowel obstruction

## Abstract

Gallstone ileus is a rare but serious complication of biliary disease that causes a mechanical obstruction in the gastrointestinal tract. Diagnosing gallstone ileus can be challenging, and if not managed quickly, it may result in severe complications. Due to frequent delays in diagnosis, this condition is associated with high morbidity and mortality rates. We report the case of a 53-year-old man who presented with right upper quadrant abdominal pain and constipation. The initial computed tomography (CT) scan revealed that these symptoms were due to a large bowel obstruction in the proximal sigmoid colon. Although a flexible sigmoidoscopy was scheduled, the patient spontaneously passed the stone in his stool before the procedure. The gallstone measured 5.5 cm x 3.9 cm, exceeding the average size of 2-5 cm reported in previous studies and ranking among the largest stones documented in gallstone ileus cases. Moreover, the size of the stone in our case exceeded the largest gallstone previously reported to pass with conservative measures in that sample. This case is notable because of the occurrence of gallstone ileus in the large bowel within an unexpected patient demographic. Furthermore, the gallstone was notably large yet passed into the stool with minimal complications.

## Introduction

Gallstone ileus is a complication of biliary disease that occurs when a gallstone causes a mechanical obstruction in the gastrointestinal tract. A gallstone often impacts the intestinal wall, forming a bilioenteric fistula between the gallbladder and gastrointestinal tract [1]. This condition was first described in 1654 by Dr. Bartholin, a Danish physician, after performing an autopsy [2]. The term *gallstone ileus* is a misnomer, as it describes a mechanical bowel obstruction caused by a gallstone rather than a functional ileus. A more accurate term would be *gallstone intestinal obstruction* [2].

This process is often caused by multiple episodes of cholelithiasis or acute cholecystitis, which causes inflammation and increased pressure within the gallbladder [1]. Over time, this pressure leads to the breakdown and ischemia of the gallbladder wall, ultimately allowing a gallstone to pass into the intestines [3]. Most gallstone ileus cases are caused by cholecystoduodenal fistulas, which account for 32.5% to 96.5% of cases [4]. An abnormal fistulous connection between the gallbladder and the colon represents a less common form of bilioenteric fistula. These rare cholecystocolonic fistulas account for only about 5% of such cases [5]. Of all gallstone ileus cases, gastrointestinal obstruction of the colon represents the rarest location and is found in 2.5%-8.1% of cases [5,6]. Imaging plays a crucial role in diagnosing gallstone ileus, with computed tomography (CT) being the preferred method for detecting Rigler’s triad: pneumobilia, an ectopic gallstone, and bowel obstruction [5,7]. Despite advancements in modern medicine, gallstone ileus continues to be of clinical concern because the mortality remains relatively high, with rates ranging from 12% to 27% [1]. 

The likelihood of spontaneous passage of a gallstone depends primarily on its size; however, the shape, location, and patient’s bowel motility are also important factors. Gallstones smaller than 5.0 mm usually pass spontaneously and do not cause gastrointestinal obstruction [6]. Stones larger than 2.5 cm in diameter rarely pass spontaneously and are most commonly impacted in the small intestine, particularly at the ileocecal valve, the narrowest portion of the gastrointestinal tract [6]. In this case, a 53-year-old male presented with a gallstone ileus of the large intestine due to a cholecystocolonic fistula; however, he was able to pass the large 5.5 cm stone without surgical intervention spontaneously.

## Case presentation

The patient was a 53-year-old male with a past medical history of recurrent nephrolithiasis, hyperlipidemia, hypertension, and prediabetes. He presented with right upper quadrant abdominal pain and constipation, which were initially attributed to his known conditions but later found to be due to gallstone ileus. Two weeks before presenting, the patient experienced diarrhea, myalgia, nausea, and emesis. One week before the presentation, he developed constant abdominal distension, pain, and constipation. During this time, he had minimal bowel movements for a week, and when able to pass stool, the patient described them as “a few pellets.” At-home remedies, such as constipation medications and enemas, were unsuccessful. The patient said he had lost eight pounds in the previous three weeks. He denied fever, chest pain, shortness of breath, chills, hematemesis, hematochezia, and tarry stools. The patient had a body mass index of 25 kg/m^2^. The patient had a regular diet. There was no past medical history or family history of sickle cell disease. Ceftriaxone and amoxicillin/clavulanic acid were initiated as empiric antibiotic therapy.

**Figure 1:**
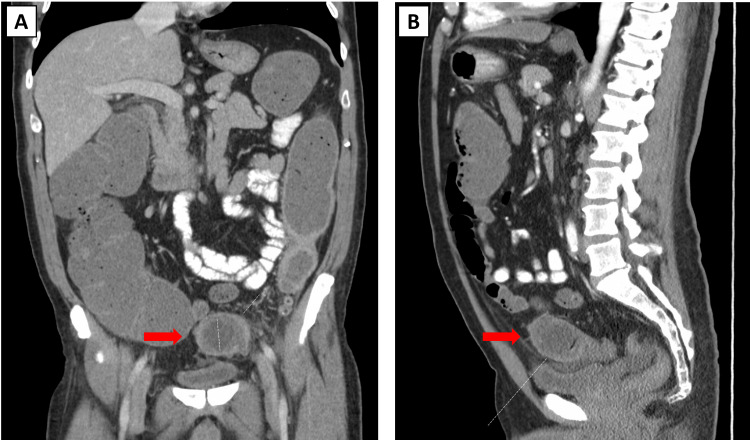
CT scan of the abdomen showing the gallstone: a round, non-calcified hypodense structure with eccentric air impacted in the proximal sigmoid colon. Red arrows indicate the gallstone: (A) coronal plane; (B) sagittal plane. CT, computed tomography

An initial abdominal CT scan revealed a round, non-calcified hypodense structure with eccentric air, impacted in the proximal sigmoid colon (Figures 1A, 1B). This suggested gallstone ileus as the cause of the large bowel obstruction. Reactive mural thickening and hyperemia extending to the splenic flexure were also seen at the level of the obstruction.

**Figure 2:**
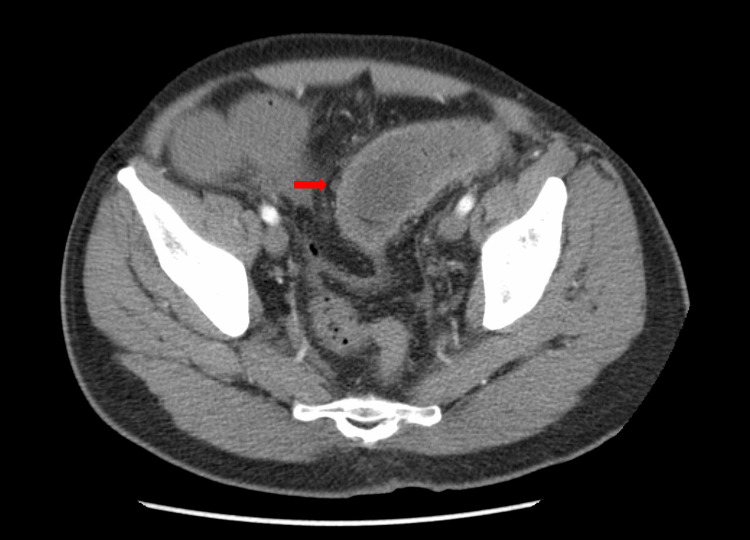
Transverse CT scan of the abdomen showing the gallstone ileus location. Reactive mural thickening and hyperemia extending to the splenic flexure were seen to the level of the obstruction. The red arrow indicates gallstone ileus. CT, computed tomography

This suggested gallstone ileus as the cause of the large bowel obstruction. Reactive mural thickening and hyperemia extending to the splenic flexure were also seen at the level of the obstruction (Figure 2). 

**Figure 3:**
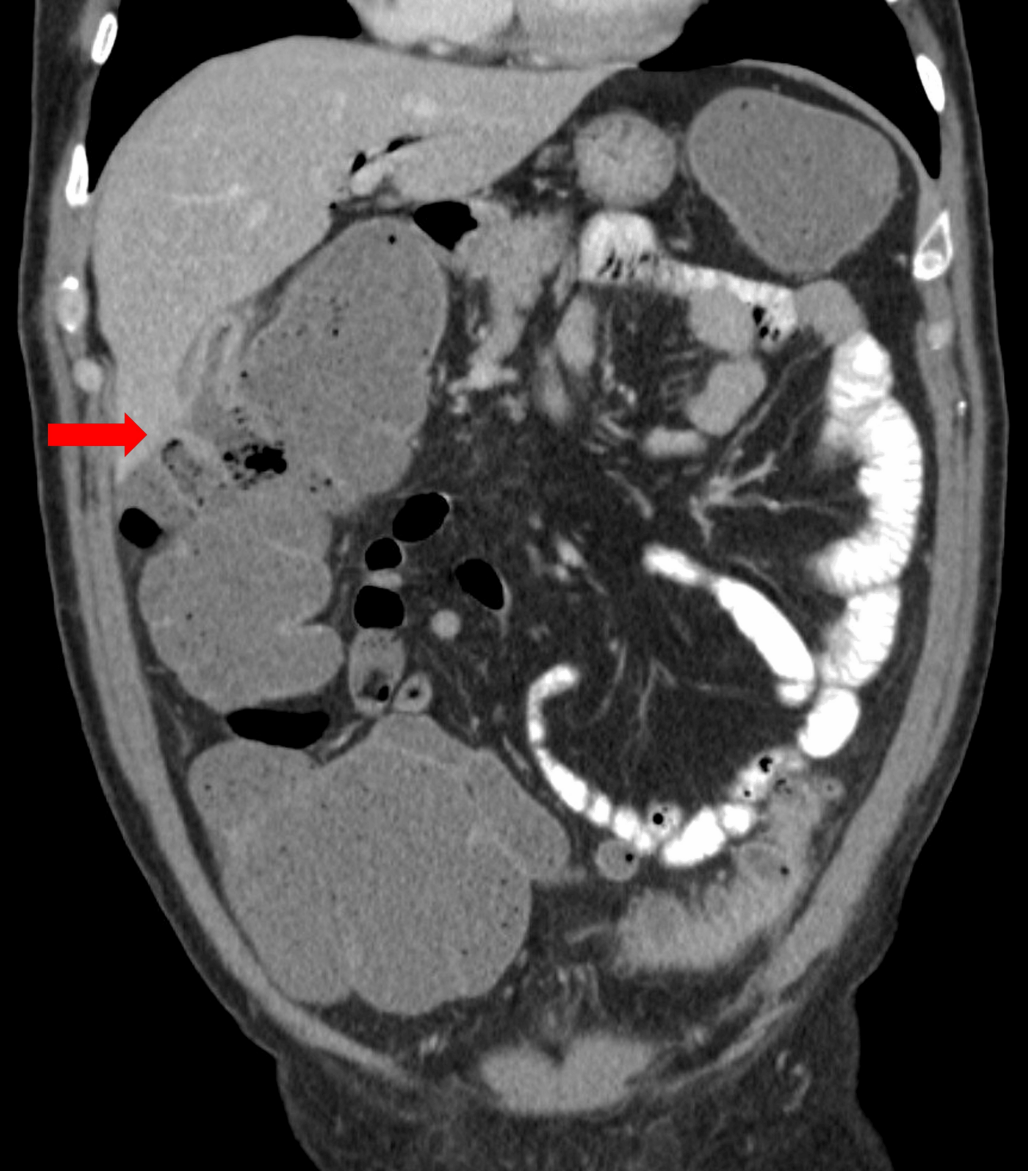
CT scan of the abdomen showing a cholecystocolonic fistula at the hepatic flexure. The red arrow indicates a cholecystocolonic fistula. CT, computed tomography

Additionally, there was pneumobilia in the bile ducts, and the gallbladder was thick-walled and under-distended. A fistulous communication between the hepatic flexure and surrounding edema was visualized (Figure 3). Imaging results indicated an underlying cholecystocolonic fistula.

**Figure 4:**
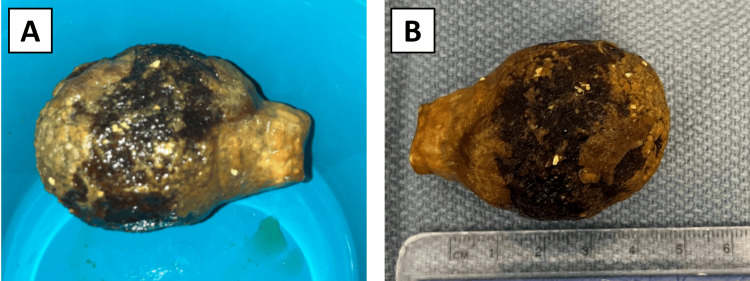
Gallstone responsible for the large bowel obstruction: (A) Gallstone after spontaneous passage; (B) measurement of the gallstone.

The patient was scheduled for a flexible sigmoidoscopy for stone retrieval, but before the procedure, he passed a large 5.5 cm x 3.9 cm gallstone in his stool (Figures 4A, 4B).

**Figure 5:**
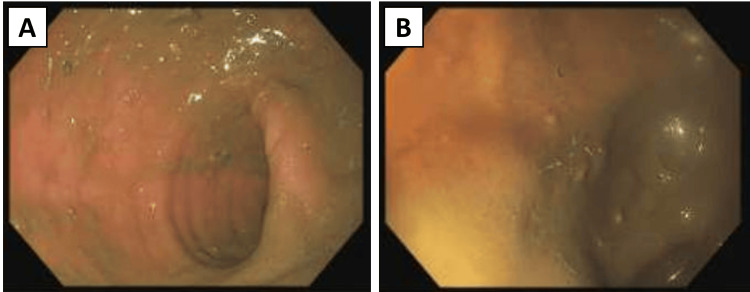
Flexible sigmoidoscopy findings after gallstone passage, confirming no additional gallstones in the lower large intestine: (A) sigmoid colon; (B) rectum.

After the gallstone passed, a flexible sigmoidoscopy confirmed that no gallstones remained in the sigmoid colon or rectum (Figures 5A, 5B). The patient was subsequently advised to undergo a cholecystectomy and start statin therapy.

**Figure 6:**
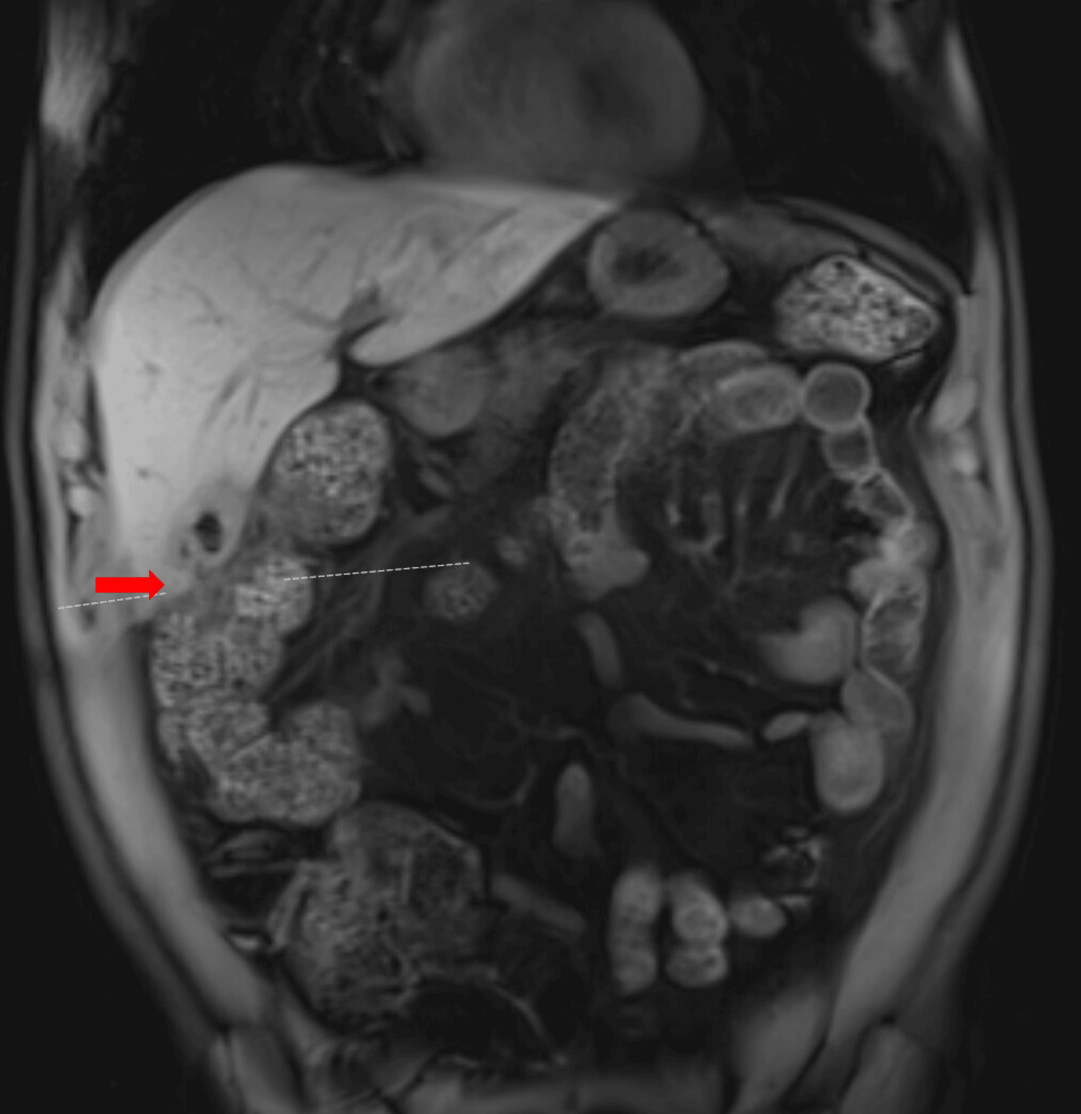
MRI of the gallbladder showing a cholecystocolonic fistula at the hepatic flexure. The gallbladder was thick-walled and hyperemic. Red arrow: cholecystocolonic fistula. MRI, magnetic resonance imaging;

A follow-up magnetic resonance imaging (MRI) scan revealed a thick-walled, hyperemic gallbladder containing a few gallstones, with a fistulous connection to the hepatic flexure (Figure 6). However, there were no suspicious gallbladder masses, and no additional obstructions were noted.

## Discussion

Gallstone ileus occurs in only 0.3%-0.5% of patients with cholelithiasis and accounts for 1%-5% of cases of all mechanical bowel obstruction [4]. The incidence of gallstone ileus is higher in females and elderly patient populations [6]. In patients over 65 years of age, gallstone ileus accounted for up to 25% of all cases of intestinal obstruction [5]. Our patient, a 53-year-old male, did not fall into the classic at-risk categories, making this case particularly noteworthy.

The symptoms of gallstone ileus are often subtle and nonspecific, making it difficult to diagnose. The onset is typically gradual, and the clinical presentation can be vague, delaying diagnosis. Initially, these symptoms may be mistaken for other gastrointestinal conditions, which can delay diagnosis. Common symptoms include nausea, vomiting, colicky abdominal pain, and abdominal distension [1,5]. However, these symptoms are indeterminate and nonspecific, making it difficult to diagnose the condition promptly. For example, nausea, vomiting, and abdominal distension are common to many gastrointestinal disorders, complicating the identification of gallstone ileus as the underlying cause. A hallmark feature of gallstone ileus is the intermittent episodes of colicky pain, known as the *tumbling phenomenon*, which occurs as the gallstone moves through the gastrointestinal tract [4]. However, colicky abdominal pain is not unique to this condition and can occur in various other abdominal issues.

Given the nonspecific nature of these symptoms, it is essential to differentiate gallstone ileus from other causes of bowel obstruction. For example, gossypiboma, a retained foreign body following surgery, often presents with similar symptoms to gallstone ileus, such as abdominal pain, vomiting, and distension. Radiologically, gossypiboma may appear as soft-tissue masses with spotted calcifications, resembling a gallstone. In a review of the literature, a case report highlighted how a retained surgical sponge led to abdominal pain, closely mimicking gallstone ileus [8]. Given these similar clinical presentations, conducting a thorough patient history and utilizing radiological imaging is imperative to distinguish between conditions and establish an accurate diagnosis.

A diagnosis of gallstone ileus is most often definitively confirmed with a CT scan of the abdomen [5]. On radiographic evaluation, Rigler's triad - pneumobilia, an ectopic gallstone, and small bowel obstruction - is pathognomonic for gallstone ileus [7]. The ileum is the most common site of gallstone impaction in gallstone ileus, followed by the jejunum and, less frequently, the duodenum [9]. These sites, all located within the small bowel, represent the primary areas affected by impaction in this condition. However, our case presents a notable deviation, as it involves a large bowel obstruction rather than the more typical small bowel obstruction.

Regarding treatment, the literature recommends initiating intravenous (IV) antibiotics and fluid resuscitation as the first steps [9]. Management from this point varies widely and is frequently tailored to the patient’s clinical presentation, comorbidities, and the medical expertise of the treating physician. If the patient is stable, conservative management can often be utilized. In these cases, the gallstones are usually smaller than 2.5 cm, and there is only a partial bowel obstruction [10].

For larger stones or complicated presentations, the literature outlines various interventions for relieving gallstone obstruction. These include advanced techniques like shockwave lithotripsy, neodymium: yttrium-aluminum-garnet (Nd: YAG) laser lithotripsy, and minimally invasive options such as endoscopic removal via colonoscopy or upper endoscopy [10]. Despite these alternatives, surgical intervention remains the most common approach [9]. Surgical interventions are often utilized to treat gallstone ileus, with several options depending on the clinical scenario. There were three primary surgical modalities used for the treatment of gallstone ileus: (1) enterolithotomy alone, (2) enterolithotomy with cholecystectomy and fistula closure (the *one-stage procedure*), and (3) enterolithotomy followed by a delayed cholecystectomy, typically four to six weeks later (the *two-stage procedure*) [4,6,11].

Among the available treatment options, colonic gallstone ileus is often treated through a surgical approach. Enterolithotomy, a procedure involving the surgical removal of the impacted gallstone by making an incision directly into the colon, is commonly performed [5]. In some cases, depending on the location of the stone and the extent of any surrounding tissue damage or infection, the surgeon may opt to perform a segmental resection of the colon. Once the stone is successfully removed, the surgeon carefully closes the incision to restore normal bowel function, taking precautions to prevent postoperative complications such as leaks or infections. In more complex cases where severe bowel obstruction, extensive inflammation, necrosis, or a risk of perforation is present, a partial colectomy may be necessary. In these instances, a Hartmann’s procedure might be performed, allowing the surgeon to remove the damaged section of the colon without the risk of contaminating the anastomosis site due to a potential leak [5]. Minimally invasive techniques may not be suitable in these situations, making more extensive surgical intervention the best option for patient care. The management of colonic gallstone ileus is particularly challenging due to the common risk factors associated with the condition, such as advanced age and severe comorbidities, which often render patients poor candidates for complex surgery.

Da Cunha et al. identified 57 cases of colonic gallstone ileus, with the sizes of the gallstones ranging from as small as 2 cm to as large as 7 cm [5]. Of these colonic cases, the mean age was 80 years old, 74% were women, and the most commonly reported symptoms were abdominal pain (84%), vomiting (67%), and constipation (61%) - two of which our patient exhibited [5]. In these cases, the gallstone was located in the sigmoid, descending sigmoid junction, or rectosigmoid junction 86% of the time [5]. The primary diagnostic modality was using a CT scan of the abdomen (81%), while laparotomy (7%), abdominal X-ray (5%), endoscopy (5%), and gastrografin (2%) were used to a lesser extent [5]. Seventy percent of patients were treated with laparotomy, while 32 underwent attempted colonoscopy or endoscopy, of which only eight were successful. Five patients received conservative treatment, and among those who experienced spontaneous evacuation, the largest stone measured 4 cm. Minimally invasive laparoscopic techniques were used in only two cases, further highlighting the rarity of using such methods for gallstone ileus treatment. Despite some successful cases using minimally invasive approaches such as laparoscopy and endoscopy/colonoscopy, these results illustrate the complexity and limitations of treating colonic gallstone ileus using less invasive techniques [5].

Gallstone ileus typically occurs in women in their sixth to eighth decades of life, with a notable gender disparity reflected in a 3:1 female-to-male ratio [1,5]. However, our patient was a male in his early 50s, falling outside the typical demographic. Moreover, he had no prior history of cholelithiasis or gallbladder disease before this incident. This case highlights the potential for the spontaneous passage of large gallstones in gallstone ileus, challenging the assumption that larger stones always require surgical intervention. While surgical removal is often the definitive treatment, there is no consensus on the preferred procedure. In the literature, gallstone ileus was caused by stones that ranged from 2 to 5 cm, with an average size of 4.3 cm for impacting stones [6]. Stones smaller than 2.5 cm can pass through the colon; the largest gallstone to pass spontaneously reported in the literature measured 5.0 cm [6,12]. This case is notable because the stone was measured at 5.5 cm and spontaneously passed through [3]. Overall, this case highlights the importance of individualized management strategies and emphasizes the need for close monitoring due to the potential outcome of a spontaneous passage. However, given the rarity of spontaneous passage of a large gallstone, further research should be conducted to identify factors that could help predict these cases.

## Conclusions

In this case, the patient spontaneously passed a 5.5 cm x 3.9 cm gallstone that was causing the obstruction and avoided the need for surgical intervention. This case underscores the importance of considering gallstone ileus in atypical populations, such as younger males. This demonstrates that even large stones can pass spontaneously, although surgical intervention remains the mainstay of treatment. It is essential to consider gallstone ileus in patients who do not fit the usual demographic profile of an older female patient. Gallstone ileus represents an uncommon cause of mechanical bowel obstruction and is often associated with high mortality rates. Because of this, while spontaneous passage of the stone is possible, physicians should still maintain a high level of suspicion in patients presenting with vague abdominal symptoms.

Each case requires individualized management. Surgical intervention remains the primary treatment for gallstone ileus, often involving enterolithotomy, cholecystectomy, or fistula closure, depending on the patient’s presentation. Alternative methods, such as minimally invasive laparoscopic techniques, endoscopy, or colonoscopy, are feasible, but there is limited literature on their success rates and viability. Patients can spontaneously pass their gallstones, relieving their obstruction without procedural intervention; however, these outcomes are an exception, and all patients should be monitored and treated promptly after diagnosis. After treatment or passage, ongoing follow-up and imaging are essential to monitor for recurrence and assess whether the chosen treatment was curative.
